# Prospective evaluation of sport activity and the development of femoroacetabular impingement in the adolescent hip (PREVIEW): results of the pilot study

**DOI:** 10.1186/s40814-022-01164-3

**Published:** 2022-09-08

**Authors:** Olufemi R. Ayeni, PREVIEW Pilot Investigators, Pierre-Olivier Jean, Nicole Simunovic, Andrew Duong, Gary Foster, Volker Musahl, Yan Sim, Lehana Thabane, Callum MacLeay, Matthew Skelly, Ajay Shanmugaraj, Dana Ghanem, Diane Heels-Ansdell, Lisa Buckingham, Vasco V. Mascarenhas, Andrea Ponniah, Etienne L. Belzile, Rintje Agricola, Seung-Hoon Baek, Hoseok Lee, Ae-Sun Chang

**Affiliations:** 1 Division of Orthopaedic Surgery, Department of Surgery, McMaster University, Hamilton, Ontario Canada; 2 McMaster University Medical Centre, 1200 Main St West, 4E15, Hamilton, Ontario L8N 3Z5 Canada

**Keywords:** Femoroacetabular impingement, Adolescents, Sports specialization, Magnetic resonance imaging

## Abstract

**Background:**

The purpose of this pilot study was to validate the feasibility of a definitive study aimed at determining if high-intensity physical activity during adolescence impacts the development of femoroacetabular impingement (FAI).

**Methods:**

This prospective cohort pilot study had a sample size target of 50 volunteers between 12 and 14 years old at sites in Canada, South Korea, and the Netherlands. Participants were evaluated clinically and radiographically at baseline and at 2 years. The participants’ sport and physical activity were evaluated using the Habitual Activity Estimation Scale (HAES) and the American Orthopaedic Society for Sports Medicine (AOSSM) criteria for sport specialization. The primary outcome was feasibility and secondary outcomes included the incidence of radiographic FAI and hip range of motion, function (Hip Outcome Score, HOS), and quality of life (Pediatric Quality of Life questionnaire, PedsQL) at 24 months. Study groups were defined at the completion of follow-up, given the changes in participant activity levels over time.

**Results:**

Of the 54 participants enrolled, there were 36 (33% female) included in the final analysis. At baseline, those classified as highly active and played at least one organized sport had a higher incidence of asymptomatic radiographic FAI markers (from 6/32, 18.8% at baseline to 19/32, 59.4% at 24 months) compared to those classified as low activity (1/4, 25% maintained at baseline and 24 months). The incidence of radiographic FAI markers was higher among sport specialists (12/19, 63.2%) compared to non-sport specialists (8/17, 47.1%) at 24 months. The HOS and PedsQL scores were slightly higher (better) among those that were highly active and played a sport compared to those who did not at 2 years (mean difference (95% confidence interval): HOS-ADL subscale 4.56 (− 7.57, 16.70); HOS-Sport subscale 5.97 (− 6.91, 18.84); PedsQL Physical Function 7.42 (− 0.79, 15.64); PedsQL Psychosocial Health Summary 6.51 (− 5.75, 18.77)).

**Conclusion:**

Our pilot study demonstrated some feasibility for a larger scale, definitive cohort study. The preliminary descriptive data suggest that adolescents engaged in higher levels of activity in sports may have a higher risk of developing asymptomatic hip deformities related to FAI but also better quality of life over the 2-year study period.

**Supplementary Information:**

The online version contains supplementary material available at 10.1186/s40814-022-01164-3.

## Key messages regarding feasibility

- There were uncertainties regarding the feasibility of recruiting and following healthy volunteer participants for 2 years.
- Participants were recruited over 12 months with a 10.4% loss to follow-up rate, with complete outcomes data, including study questionnaires and MRIs, collected for 75% (36/48) of participants at 2 years.
- These feasibility findings led to protocol changes for the main study including involving additional international sites, implementing novel recruitment strategies, adding interim follow-up visits every 3 months, and introducing an activity log and wrist-worn tracker to improve the collection of physical activity data over the 2-year period.

## Introduction

Femoroacetabular impingement (FAI) is a relatively newly recognized painful hip condition. The development of pain in this manner serves as an indicator of early damage to the hip and FAI has been implicated as the probable principal cause of idiopathic hip osteoarthritis (OA) later in life [1–5]. It is classically described as impingement between the femoral head and neck (FHN) junction and acetabular rim because of bony deformities in one or both of these regions [6]. Therefore, two morphologies have been classified: cam morphology—defined as femoral head asphericity or FHN convexity; and pincer morphology—where there is an overcoverage of the femoral head by the acetabulum. It is estimated that most cases of FAI are a mix of both cam and pincer-type morphology [3, 7].

Prior literature has estimated that FAI prevalence ranges from 14 to 23% in the asymptomatic general population and up to 95% in athletes [8–11]. Awareness of FAI among clinicians has caused a rise in its diagnosis across all age categories but more so in pediatric/adolescent populations [12]. Suggested causes of FAI have included genetics, subclinical hip disease, and stresses to the hip joint from repetitive and high intensity activities at a younger age [13]. According to Packer et al., the genetic implication is not clear, and in otherwise healthy children, there is growing evidence that participation in high level athletics during adolescence causes a higher prevalence of FAI, especially cam morphology [14].

High-impact activities, especially during physeal closure, have been shown to have the potential to cause hip damage [15, 16]. More specifically, high-impact activities have been shown to affect the development of the femur at the growth plate region and FHN junction [17, 18]. Children can suffer more consequences of injuries and shear forces that can cause premature physeal closure, apophyseal avulsion, and chondral injuries because of their open physes and growing cartilage [19]. Typical activities that have been implicated in FAI development include soccer, ice hockey, basketball, and football [10, 17, 20–22]. It has been posited that there is a dose-response relationship between the amount of hours of practice every week during adolescence and the eventual incidence of cam-type morphology in adulthood among athletes [15]. This is certainly of concern given the increasing trend toward year-round participation in youth sports with early specialization [23–25]. Consequently, research is needed to determine “how much is too much” sport activity in order to advocate for the young who cannot easily protect themselves from excesses [26].

As FAI is diagnosed more frequently in athletes, and with over 38 million young athletes participating in organized sports in the USA alone [19], it has become a priority to identify modifiable variables in order to mitigate the risk of developing FAI in children and adolescents. This is especially important due to the link between FAI and hip OA later in life [12]. The PREVIEW pilot study was initiated to evaluate the feasibility of a large-scale prospective cohort to evaluate the potential risk of developing FAI-related hip deformities based on the intensity, duration, and specialization of sport activity during adolescence.

## Methods

### Study design and participant selection

This study was a prospective cohort pilot with a sample size target of 50 healthy volunteers between the ages of 12–14 years. Participants were excluded if they had a closed physeal plate (i.e., a mature hip), hip complaints at the time of enrollment, previous hip trauma or surgery, any medical comorbidity that would limit activities of daily living (ADLs), any history of or ongoing pediatric hip disease, or were unwilling to undergo a magnetic resonance imaging (MRI). MRI was used because it does not involve any radiation. Participants were recruited by experienced hip surgeons and sports medicine researchers at 4 clinical sites in Canada (2), South Korea (1), and the Netherlands (1). All participating sites obtained ethics approval. Participants were evaluated clinically and radiographically at baseline and at 2 years.

### Defining activity level and sport specialization

The participants’ duration and intensity of any sport and general physical activity were evaluated using the self-reported Habitual Activity Estimation Scale (HAES) and sport specialization was classified according to the American Orthopaedic Society for Sports Medicine (AOSSM) criteria [27]. The HAES quantifies the duration, in hours, of 4 levels of activity ranging from very inactive (lying down, napping), somewhat inactive (sitting, reading, watching television, playing video games), somewhat active (walking, light chores), to very active (running, bicycling, activities leading to sweating or breathing hard) over a complete waking day [28]. The cut-off of 180 min in a given category was used according to prior sport specialization criteria and plans for the definitive trial [29]. The HAES is designed for administration in the pediatric population and has been shown to be both a valid and reliable form of quantifying levels of physical activity [28]. Participants classified as “highly active” included those that scored “very active” on the HAES and reported playing at least one sport. Using the AOSSM criteria, a sport specialist was defined as someone who participated in intensive (i.e., HAES = “very active”) training and/or competition in one sport greater than 8 months per year [27]. Study groups were defined at the completion of follow-up given participant activity levels were expected to change over time.

### Objectives and hypotheses

The primary outcome was to determine the feasibility of a large-scale prospective cohort study, based on the following thresholds established a priori: (1) 50 subjects recruited within 3 months, (2) 45 of 50 participants (90%) achieving full follow-up at 2 years, and (3) completion of at least 90% of all outcome measures including the Hip Outcome Score (HOS), Pediatric Quality of Life (PedsQL) questionnaire, hip MRI, and range of motion (ROM).

Secondary outcomes included the incidence of radiographic FAI as determined by a blinded, independent evaluation of an MRI of the dominant hip (planned primary outcome of the definitive study), ROM, hip function evaluated using the HOS, and health-related quality of life (HRQL) evaluated using the PedsQL questionnaire at 24 months. An additional secondary objective was to determine if the presence of FAI morphology at baseline was associated with differences in participant-reported hip function and HRQL at 24 months. We hypothesized that (1) participants engaging in specialized, high intensity sport activity would have a higher prevalence of FAI morphology diagnosed through MRI compared to non-sport specialists at varying activity levels in the same age group at 2 years; (2) participants diagnosed with any type of FAI (at baseline) would have decreased hip function and HRQL at 2 years compared to those without; and (3) participants with symptomatic FAI would have lower functional and HRQL scores at 2 years compared to participants that did not have FAI morphology or were asymptomatic.

### Outcomes

Clinical assessments at baseline and 2 years consisted of a hip ROM evaluation and response to both anterior and posterior hip impingement tests, as well as log roll test. We used a non-contrast 3D-volumetric interpolated breath-hold examination (VIBE) sequence for each MRI [30]. An independent, blinded adjudicator (a senior radiologist with more than 20 years of experience) evaluated all MRIs for FAI morphology using the criteria shown in Table 1. The scores for the individual domains for the HOS (ADL and sports subscale) and PedsQL (physical functioning, emotional functioning, social functioning, school functioning, psychosocial health summary) are presented [31–35].

**Table 1:** Criteria for defining FAI

FAI type	Criteria
Cam	☐ Alpha angle is > 55° ☐ Anterior head-neck offset < 10 mm ☐ Anterior head-neck offset ratio < 0.15 *Must meet 2/3 criteria to be considered cam impingement
Pincer	☐ Acetabular depth < 3 mm ☐ Lateral center-edge angle >39° ☐ Acetabular anteversion (in the upper third of the femoral head) < 0° *Must meet 2/3 criteria to be considered pincer impingement
Mixed	*Must meet 2/3 criteria each for cam and pincer impingement (as per above) to be considered mixed impingement

### Statistical analysis

Given this was a pilot study with a small sample size, the analyses were exploratory and descriptive. We present the data using means, mean difference (MD), and proportions with standard deviations and 95% confidence intervals (CI). All outcomes presented are based on 24-month data. All analyses were conducted using SAS version 9.4 (Cary, NC).

## Results

Of the 54 participants enrolled, there were 36 included in the final analysis, 66.7% of which were male and the mean age was 13.61 (± 0.81) years. Eighteen participants were excluded: 7 had incomplete data sets, 6 had a closed physeal plate identified at the baseline MRI, and 5 were lost to follow-up. The 6 participants with a closed physeal plate were all female and ranged in age between 12.9 and 14.5 years and activity level from none (2/6), to light (1/6), to vigorous (3/6) at the baseline evaluation. The majority of participants had a normal BMI, no comorbidities, and reported their baseline sport activity as “vigorous.” On average, participants played one or more sports for 3.53 (± 2.06) hours per week. The most commonly played sports among the participants in this study included soccer (44.4%), hockey (25.0%), and basketball (16.7%) (Table 2). Based on the AOSSM criteria, a total of 19 participants were categorized as sport specialists and 17 as non-sport specialists (Table 2). When grouped according to activity level, there were 32 participants classified as highly active who played at least one sport and 4 as low activity who played minimal or no sports (Additional Table 1).

**Table 2:** Participant demographics by sport specialization

	Sport specialists ***N*** = 19	Non-sport specialists ***N*** = 17	Total ***N*** = 36
Age, mean (SD)	13.40 (0.63)	13.85 (0.94)	13.61 (0.81)
Gender, *n* (%)			
Male	16 (84.2)	8 (47.1)	24 (66.7)
Female	3 (15.8)	9 (52.9)	12 (33.3)
Ethnicity, *n* (%)			
Asian	15 (79.0)	5 (29.4)	20 (55.6)
White/Caucasian	4 (21.1)	12 (70.6)	16 (44.4)
BMI, *n* (%)			
< 18.5 (underweight)	8 (42.1)	8 (47.1)	16 (44.4)
18.5–24.9 (normal weight)	10 (52.6)	8 (47.1)	18 (50.0)
25–29.9 (overweight)	0 (0)	1 (5.9)	1 (2.8)
30–39.9 (obese)	1 (5.3)	0 (0)	1 (2.8)
Dominant hip, *n* (%)			
Left	1 (5.3)	3 (17.7)	4 (11.1)
Right	18 (94.7)	14 (82.4)	32 (88.9)
Co-morbidities, *n* (%)			
None	15 (79.0)	10 (58.8)	25 (69.4)
Allergic rhinitis	1 (5.3)	1 (5.9)	2 (5.6)
Asthma	1 (5.3)	1 (5.9)	2 (5.6)
Atopic dermatitis	0 (0)	1 (5.9)	1 (2.8)
Attention deficit	0 (0)	1 (5.9)	1 (2.8)
Attention deficit with hyperactivity	0 (0)	1 (5.9)	1 (2.8)
Previous lower extremity injury	1 (5.3)	1 (5.9)	2 (5.6)
Rhinitis	2 (10.5)	0 (0)	2 (5.6)
Scoliosis	0 (0)	1 (5.9)	1 (2.8)
Baseline sport activity, *n* (%)			
Light	0 (0)	2 (11.8)	2 (5.6)
Moderate	1 (5.3)	4 (23.5)	5 (13.9)
Vigorous	18 (94.7)	11 (64.7)	29 (80.6)
Hours per week playing sports, mean (SD)	3.32 (1.83)	3.76 (2.33)	3.53 (2.06)
Sports played, *n* (%)			
Soccer	7 (36.8)	9 (52.9)	16 (44.4)
Hockey	3 (15.8)	6 (35.3)	9 (25.0)
Basketball	1 (5.3)	5 (29.4)	6 (16.7)
Racquet sports	3 (15.8)	2 (11.8)	5 (13.9)
Volleyball	2 (10.5)	3 (17.7)	5 (13.9)
Skiing/snowboard	0 (0)	5 (29.4)	5 (13.9)
Archery	3 (15.8)	0 (0)	3 (8.3)
Multiple sports	0 (0)	3 (17.7)	3 (8.3)
Cycling	0 (0)	2 (11.8)	2 (5.6)
Football	0 (0)	2 (11.8)	2 (5.6)
Running (long)	0 (0)	2 (11.8)	2 (5.6)
Running (short)	0 (0)	2 (11.8)	2 (5.6)
Swimming	0 (0)	2 (11.8)	2 (5.6)
Equestrian	0 (0)	1 (5.9)	1 (2.8)
Gymnastics	0 (0)	1 (5.9)	1 (2.8)
Lacrosse	0 (0)	1 (5.9)	1 (2.8)
Weightlifting	0 (0)	1 (5.9)	1 (2.8)
Frisbee	0 (0)	1 (5.9)	1 (2.8)
Hiking	0 (0)	1 (5.9)	1 (2.8)

### Feasibility measures

The investigators recruited 54 participants in 12 months and of those 48 eligible (i.e., with open physeal plates) the loss to follow-up rate was 10.4% (5/48) with complete outcomes data, including study questionnaires and MRIs, collected for 75% (36/48) of participants at 2 years. Of the 5 participants lost to follow-up, 2 could not be contacted and 3 refused to return for their 2-year MRI due to fears/restrictions related to coming into the hospital during the COVID-19 pandemic.

### Other outcome measures

At baseline, those classified as highly active and played at least one organized sport had a higher incidence of asymptomatic radiographic FAI markers (from 6/32, 18.8% at baseline to 19/32, 59.4% at 24 months) compared to those classified in the low activity, with or without playing a sport group (1/4, 25% maintained at baseline and 24 months). The incidence of radiographic FAI markers was higher among sport specialists (12/19, 63.2%) compared to non-sport specialists (8/17, 47.1%) at 24 months. There was one participant with pincer-type FAI in the high activity/sport specialist group while all other cases were cam-type. All participants with FAI morphology were asymptomatic.

Mean hip ROM measures were slightly reduced from baseline to 2 years across all activity levels (Table 3). The MRI measurements revealed there was a slightly higher proportion of labral tears in the highly active group (57%, 16/28) compared to the low activity group (50%, 1/2) at 24 months. Overall, among participants with radiographic FAI markers at 24 months, 43.3% (13/30) had a labral tear. Partial thickness cartilage lesions were present in 25% of participants 24 months (Table 4).

**Table 3:** Hip range of motion measurements by activity level

	High activity with sports		Low activity with or without sports	
	Baseline ***N*** = 32	24 months ***N*** = 29	Baseline ***N*** = 4	24 months ***N*** = 4
Anterior impingement test, *n* (%)				
Left hip				
Positive	0 (0)	1 (3)	0 (0)	0 (0)
Negative	32 (100)	28 (97)	4 (100)	4 (100)
Right hip				
Positive	0 (0)	2 (7)	0 (0)	0 (0)
Negative	32 (100)	27 (93)	4 (100)	4 (100)
Posterior impingement test, *n* (%)				
Left hip				
Positive	0 (0)	0 (0)	0 (0)	0 (0)
Negative	32 (100)	29 (100)	4 (100)	4 (100)
Right hip				
Positive	1 (3)	1 (3)	0 (0)	0 (0)
Negative	31 (97)	28 (97)	4 (100)	4 (100)
Log roll test, *n* (%)				
Left hip				
Positive	0 (0)	0 (0)	0 (0)	0 (0)
Negative	32 (100)	29 (100)	4 (100)	4 (100)
Right hip				
Positive	0 (0)	0 (0)	0 (0)	0 (0)
Negative	32 (100)	29 (100)	4 (100)	4 (100)
Left hip range of motion, mean (SD)				
Flexion	127.38 (10.13)	125.86 (8.67)	127.75 (6.85)	123.75 (11.09)
Extension	31.50 (6.55)	29.03 (7.65)	27.75 (3.86)	27.50 (8.66)
Abduction	53.56 (14.42)	59.90 (14.38)	50.00 (15.14)	51.25 (13.15)
Adduction	33.72 (8.68)	32.41 (7.27)	33.25 (4.99)	26.25 (7.50)
Internal rotation (neutral)	40.03 (15.73)	37.86 (11.68)	48.75 (6.99)	41.25 (14.93)
External rotation (neutral)	59.06 (12.39)	54.03 (14.97)	63.50 (9.43)	48.75 (22.87)
Internal rotation (90° flexion)	34.13 (10.86)	32.86 (13.67)	34.50 (13.96)	30.00 (17.32)
External rotation (90° flexion)	57.69 (16.59)	57.93 (16.77)	50.00 (24.51)	52.5 (22.17)
Right hip range of motion, mean (SD)				
Flexion	126.19 (10.17)	124.93 (9.05)	129.75 (6.85)	121.25 (10.31)
Extension	31.06 (6.06)	28.10 (7.72)	28.00 (2.45)	27.50 (8.66)
Abduction	53.84 (14.28)	58.10 (13.52)	54.00 (15.51)	53.75 (13.77)
Adduction	33.25 (7.80)	32.93 (8.19)	31.75 (2.36)	27.50 (9.57)
Internal rotation (neutral)	40.22 (14.6)	35.24 (13.09)	43.75 (17.02)	40.00 (13.54)
External rotation (neutral)	58.50 (13.)	55.86 (14.88)	54.25 (18.30)	52.50 (23.27)
Internal rotation (90° flexion)	34.56 (11.73)	32.48 (13.74)	43.25 (10.72)	30.00 (17.32)
External rotation (90° flexion)	58.78 (17.01)	58.48 (14.53)	56.00 (21.42	51.25 (21.75)

**Table 4:** Hip MRI measurements by activity level

	High activity with sports		Low activity with or without sports	
	Baseline ***N*** = 32	24 months ***N*** = 28	Baseline ***N*** = 4	24 months ***N*** = 4
Diameter of femoral head, mean (SD)	43.97 (3.39)	46.35 (3.17)	40.78 (2.32)	41.53 (2.65)
Lateral centre-edge angle, mean (SD)	24.94 (5.33)	27.71 (5.55)	30.50 (8.58)	29.00 (7.62)
Acetabular inclination angle, mean (SD)	6.84 (4.06)	7.51 (5.00)	4.43 (3.34)	5.75 (4.27)
Posterior acetabular sector angle, mean (SD)	85.89 (6.83)	90.36 (4.68)	88.63 (8.88)	91.5 (6.24)
Anterior acetabular sector angle, mean (SD)	63.7 (5.37)	60.82 (6.33)	59.68 (4.08)	55.25 (4.11)
Acetabular anteversion angle—upper third, mean (SD)	-1.68 (5.48)	1.84 (6.67)	2.50 (9.33)	3.50 (8.96)
Acetabular anteversion angle—centre, mean (SD)	11.30 (3.64)	15.04 (3.92)	15.75 (6.95)	21.00 (10.3)
Cranial acetabular retroversion, *n* (%)				
Yes	15 (47)	6 (21)	1 (25)	1 (25)
No	15 (47)	22 (79)	3 (75)	3 (75)
Acetabular depth (mm), mean (SD)	4.78 (1.60)	5.74 (1.88)	4.70 (0.48)	5.25 (0.6)
Alpha angle, mean (SD)				
12:00	45.06 (5.92)	44.99 (5.98)	43.95 (6.09)	46.25 (3.59)
1:00	49.63 (6.73)	56.48 (9.44)	48.13 (3.57)	49.18 (4.31)
1:30	52.8 (5.99)	57.64 (6.83)	51.93 (2.41)	54.18 (3.81)
2:00	52.58 (4.25)	56.54 (6.94)	52.43 (1.95)	57.13 (6.86)
3:00	48.94 (5.44)	45.57 (6.36)	47.50 (3.87)	43.00 (4.97)
Neck shaft angle, mean (SD)	132.81 (5.31)	133.04 (4.93)	133.75 (3.1)	132.5 (3.11)
Femoral offset, mean (SD)				
12:00	6.74 (1.67)	6.80 (1.41)	6.00 (1.28)	5.90 (0.75)
1:00	5.74 (1.4)	4.81 (0.94)	5.45 (0.98)	4.73 (0.68)
1:30	5.77 (1.22)	4.70 (0.92)	4.78 (0.55)	5.10 (0.62)
2:00	5.91 (1.23)	4.94 (1.26)	4.55 (0.79)	5.23 (0.46)
3:00	8.03 (1.24)	7.65 (1.61)	7.40 (1.88)	7.60 (2.05)
Femoral offset ratio, mean (SD)				
1:00	0.13 (0.03)	0.10 (0.02)	0.13 (0.03)	0.11 (0.01)
1:30	0.13 (0.03)	0.13 (0.13)	0.12 (0.01)	0.12 (0.01)
2:00	0.13 (0.03)	0.11 (0.03)	0.11 (0.02)	0.13 (0.01)
3:00	0.18 (0.02)	0.17 (0.03)	0.18 (0.04)	0.18 (0.05)
Modified MAHORN classification, *n* (%)				
Partial-thickness cartilage lesion	2 (6)	7 (25)	0 (0)	1 (25)
No cartilage lesion	28 (88)	21 (75)	4 (100)	3 (75)
Labral tears present, *n* (%)				
Yes	11 (34)	16 (57)	1 (25)	2 (50)
Anterior	7 (22)	9 (32)	0 (0)	0 (0)
Superior/lateral	4 (13)	11 (39)	1 (25)	2 (50)
No	19 (59)	12 (43)	3 (75)	2 (50)
Herniation pits present, *n* (%)				
Yes	1 (3)	5 (18)	0 (0)	1 (25)
No	29 (91)	23 (82)	4 (100)	3 (75)
FAI type, *n* (%)				
Cam	6 (19)	18 (64)	1 (25)	1 (25)
Pincer	1 (3)	1 (4)	0 (0)	0 (0)
None	25 (78)	9 (32)	3 (75)	3 (75)

The HOS and PedsQL scores were slightly higher (better) among those that were highly active and played a sport compared to those who did not at 2 years [MD (95% CI): HOS-ADL subscale 4.56 (− 7.57, 16.70); HOS-Sport subscale 5.97 (− 6.91, 18.84); PedsQL Physical 7.42 (− 0.79, 15.64); PedsQL Emotional 11.87 (− 6.74, 30.49); PedsQL Social 5.94 (− 2.86, 14.74); PedsQL School 1.72 (− 17.32, 20.76); PedsQL Psychosocial 6.51 (− 5.75, 18.77)] (Additional Table 2). There was no difference in HOS and PedsQL scores at 2 years between participants with FAI morphology at baseline compared to those without (Additional Table 3).

## Discussion

With respect to the feasibility objectives, this pilot study required a longer period of recruitment (12 months vs. target of 3 months), had a lower than expected outcomes completion rate (75% vs. target of 90%), but did meet the follow-up target of 90% for at least some 2-year data (based on the 48 participants that were eligible for the study). The available pilot data suggest that adolescents engaged in higher levels of activity in sports or who specialize in one sport may have a higher risk of developing asymptomatic FAI morphology (predominantly cam-type) at 2 years. Those in the high activity/sport specialist groups also reported higher HOS and PedsQL scores across all domains when compared to the low activity/non-sport specialist groups at 2 years. Where all participants with FAI morphology were asymptomatic, there was no difference in hip function (HOS) or HRQL (PedsQL) between those with FAI morphology at baseline compared to those without at 2 years.

Although not all of the feasibility objectives were met for this pilot study, it helped to inform important protocol changes for the definitive study which is currently underway. To address recruitment and enhance external validity, we involved additional international sites in the definitive study and implemented several important recruitment strategies including posting study flyers at each participating institution, distributing study flyers online and on paper to grades in the eligible age range at schools in the regions of the recruiting institutions, and having the co-investigators reach out to community coaches and competitive sport organizations and clubs. To promote complete data collection and improve participant retention, we added interim follow-up visits every 3 months and introduced an activity log and wrist-worn tracker to improve the collection of physical activity data over the 2-year period. The definitive study protocol has been published [29].

Although limited by a small sample size, the pilot study found that around 60% of high activity/sport specialist participants had radiographic FAI morphology at 24 months. Where approximately 20% had evidence of FAI at baseline, this a noteworthy increase as no participants in the low activity level group developed FAI. These results are similar to many cross-sectional studies that have found the prevalence of cam deformity in high impact sports to be between 26 and 89% [17, 20–22, 36, 37]. A recent longitudinal MRI study followed 25 high level (3 to 5 sessions per week) male ice hockey players aged 11 to 13 years old for 3 years and found that 50% of their cohort presented with FAI at the end of the study and that cam morphology appeared to develop at the beginning of the final growth spurt of the femoral head [18]. They also noted that four participants still had open physes at final follow-up, which could underestimate the final number of participants developing FAI [18]. A study from Agricola *et al.* followed 89 preprofessional soccer players for 2 years and found that boys aged 12 or 13 years old had a significant increase in presence of a flattened head and neck junction going from 13.6% at baseline to 50% at 24 months [38]. A study from Tak et al. found that football players who started playing before the age 12 at a high frequency had a statistically significant higher prevalence of cam deformities compared to players who started after 12 and played at lower frequency (64% vs 40%) [37].

A slightly higher percentage of participants in the highly active group presented with asymptomatic labral tears at 24 months (57% vs. 50%). It is now well accepted that cam-type FAI is a risk factor for labral tears [39]. A recent MRI study looking at young football players (mean age 26 years) found an association between cam morphology size and risk of chondrolabral damage. Interestingly, the relationship was similar in symptomatic and asymptomatic patients [40]. With chondrolabral damage being a continuum, it is likely too early for participants in this study to show any important cartilage damage that may cause hip pain [39].

While there may be an increased risk in developing FAI if playing high intensity sports in adolescence, there still appear to be benefits to quality of life and hip function. A survey and focus group study of University students and community members found that physical activity was a positive contributor to all aspects of HRQL and that emotional benefit was reported as equally important as physical health in younger participants and more important than physical health among community members [41]. Systematic reviews evaluating physical activity interventions or the impact of sedentary behavior have found that a higher prevalence of physical activity is associated with the psychological well-being of children and adolescents [42]. Interestingly, participants in the highly active group reported better hip function (according to the HOS scores) than their less active counterparts, despite having a higher proportion of radiographic FAI at 24 months. This is likely due to many factors including the fact that they were asymptomatic, had stronger hips due to increased activity, and potentially better access to training and rehabilitation. Ultimately, there has to be a balance between protecting the hip from developing FAI, while still promoting a high level of physical activity in adolescence given its other benefits.

This study had some limitations. Because this was a pilot to help determine feasibility and generate hypotheses, we were unable to make any definitive conclusions at this time due to the small sample size. We did not meet all of our pre-defined feasibility criteria but were able to adjust the protocol to help ensure the success of the definitive prospective cohort study [29]. In addition, we expect a higher follow-up rate as the definitive study continues post-COVID-19 pandemic.

## Conclusion

Our pilot study demonstrated some feasibility for a larger scale, definitive cohort study. The preliminary descriptive data suggest that adolescents engaged in higher levels of activity in sports may have a higher risk of developing asymptomatic hip deformities related to FAI, but also better HRQL over the 2-year study period. The results of this pilot were used to refine the protocol for the ongoing definitive PREVIEW study (*N* = 200, NCT03891563).

## Supplementary Information

**Additional file 1: Additional Table 1.** Participant demographics by activity level. **Additional Table 2.** Hip function and health-related quality of life outcomes by activity level. **Additional Table 3.** Hip function and health-related quality of life outcomes by baseline FAI.

## Data Availability

The datasets supporting the conclusions of this article are included within the article and its additional files.

## Abbreviations

ADLs: Activities of daily living
AOSSM: American Orthopaedic Society for Sports Medicine
CI: Confidence intervals
FAI: Femoroacetabular impingement
FHN: Femoral head and neck
HAES: Habitual Activity Estimation Scale
HOS: Hip Outcome Score
HRQL: Health-related quality of life
MD: Mean difference
MRI: Magnetic resonance imaging
OA: Osteoarthritis
PedsQL: Pediatric Quality of Life questionnaire
VIBE: Volumetric interpolated breath-hold examination
