# Supplementary material for: Prospective evaluation of sport activity and the development of femoroacetabular impingement in the adolescent hip (PREVIEW): results of the pilot study
Source: Pilot Feasibility Stud. 2022 Sep 8;8:201. doi: 10.1186/s40814-022-01164-3 (PMC9452871; doi:10.1186/s40814-022-01164-3)
Supplement: Supplementary file 1 — Additional file 1: Additional Table 1. Participant demographics by activity level. Additional Table 2. Hip function and health-related quality of life outcomes by activity level. Additional Table 3. Hip function and health-related quality of life outcomes by baseline FAI. [file 40814_2022_1164_MOESM1_ESM.docx]

**Additional Files**

**Appendix Table 1.** Participant demographics by activity level

|  | **High Activity with Sports**  **N=32** | **Low Activity with or without Sports**  **N=4** | **Total**  **N=36** |
| --- | --- | --- | --- |
| Age, mean (SD) | 13.62 (0.76) | 13.57 (1.33) | 13.61 (0.81) |
| Gender, n (%)  Male  Female | 23 (71.9)  9 (28.1) | 1 (25.0)  3 (75.0) | 24 (67)  12 (33) |
| Ethnicity, n (%)  Asian  White/Caucasian | 18 (56.3)  14 (43.8) | 2 (50.0)  2 (50.0) | 20 (56)  16 (44) |
| BMI, n (%)  <18.5 (Underweight)  18.5-24.9 (Normal weight)  25-29.9 (Overweight)  30-39.9 (Obese) | 14 (43.8)  17 (53.1)  0 (0)  1 (3.1) | 2 (50.0)  1 (25.0)  1 (25.0)  0 (0) | 16 (44)  18 (50)  1 (3)  1 (3) |
| Dominant Hip, n (%)  Left  Right | 4 (12.5)  28 (87.5) | 0 (0)  4 (100) | 4 (11.1))  32 (89) |
| Co-morbidities, n (%)  None  Allergic Rhinitis  Asthma  Atopic Dermatitis  Attention Deficit  Attention Deficit With  Hyperactivity  Previous Lower Extremity  Injury  Rhinitis  Scoliosis | 23 (71.9)  2 (6.3)  2 (6.3)  0 (0)  1 (3.1)  1 (3.1)  2 (6.3)  2 (6.3)  0 (0) | 2 (50.0)  0 (0)  0 (0)  1 (25.0)  0 (0)  0 (0)  0 (0)  0 (0)  1 (25.0) | 25 (69.4)  2 (5.6)  2 (5.6)  1 (2.8)  1 (2.8)  1 (2.8)  2 (5.6)  2 (5.6)  1 (2.8) |
| Baseline Sport Activity, n (%)*  Light  Moderate  Vigorous | 0 (0)  3 (9.4)  29 (90.6) | 2 (50.0)  2 (50.0)  0 (0) | 2 (5.6)  5 (13.9)  29 (80.6) |
| Hours Per Week Playing Sports, mean (SD) | 3.56 (2.08) | 3.25 (2.22) | 3.53 (2.06) |
| Sports Played, n (%) |  |  |  |
| Soccer | 15 (46.9) | 1 (25) | 16 (44.4) |
| Hockey | 9 (28.1) | 0 (0) | 9 (25.0) |
| Basketball | 5 (15.6) | 1 (25) | 6 (16.7) |
| Racquet Sports | 4 (12.5) | 1 (25) | 5 (13.9) |
| Volleyball | 4 (12.5) | 1 (25) | 5 (13.9) |
| Skiing/Snowboard | 4 (12.5) | 1 (25) | 5 (13.9) |
| Archery | 3 (9.4) | 0 (0) | 3 (8.3) |
| Multiple Sports | 2 (6.3) | 1 (25) | 3 (8.3) |
| Cycling | 1 (3.1) | 1 (25) | 2 (5.6) |
| Football | 2 (6.3) | 0 (0) | 2 (5.6) |
| Running (Long) | 2 (6.3) | 0 (0) | 2 (5.6) |
| Running (Short) | 2 (6.3) | 0 (0) | 2 (5.6) |
| Swimming | 1 (3.1) | 1 (25) | 2 (5.6) |
| Equestrian | 1 (3.1) | 0 (0) | 1 (2.8) |
| Gymnastics | 1 (3.1) | 0 (0) | 1 (2.8) |
| Lacrosse | 1 (3.1) | 0 (0) | 1 (2.8) |
| Weightlifting | 1 (3.1) | 0 (0) | 1 (2.8) |
| Frisbee | 1 (3.1) | 0 (0) | 1 (2.8) |
| Hiking | 0 (0) | 1 (25) | 1 (2.8) |

**Based on subjective evaluation by the participant*

**Appendix Table 2.** Hip function and health-related quality of life outcomes by activity level

| **Mean (SD)** | **Total**  **N=36** | **High Activity with Sports**  **N=32** | **Low Activity with or without Sports**  **N=4** | **Mean Difference***  **(95% CI)** |
| --- | --- | --- | --- | --- |
| HOS-ADL | 99.31 (2.72) | 99.81 (0.59) | 95.25 (7.63) | 4.56 (-7.57, 16.70) |
| HOS-Sport | 99.06 (3.15) | 99.72 (0.79) | 93.75 (8.10) | 5.97 (-6.91, 18.84) |
| PedsQL |  |  |  |  |
| Physical | 94.88 (7.88) | 95.70 (7.44) | 88.28 (9.33) | 7.42 (-0.79, 15.64) |
| Emotional | 85.56 (17.43) | 86.88 (16.35) | 75.00 (24.83) | 11.87 (-6.74, 30.49) |
| Social | 96.53 (8.27) | 97.19 (8.32) | 91.25 (6.29) | 5.94 (-2.86, 14.74) |
| School | 82.78 (17.42) | 82.97 (17.91) | 81.25 (14.93) | 1.72 (-17.32, 20.76) |
| Psychosocial Health  Summary | 88.29 (11.40) | 89.01 (11.81) | 82.50 (4.81) | 6.51 (-5.75, 18.77) |

**High activity with sports – Low activity with or without sports*

**Appendix Table 3.** Hip function and health-related quality of life outcomes by baseline FAI

| **Mean (SD)** | **Total**  **N=36** | **Participants with Baseline FAI**  **N=8** | **Participants without Baseline FAI**  **N=28** | **Mean Difference***  **(95% CI)** |
| --- | --- | --- | --- | --- |
| HOS-ADL | 99.31 (2.72) | 99.50 (1.07) | 99.25 (3.05) | 0.25 (-1.65, 1.15) |
| HOS-Sport | 99.06 (3.15) | 99.63 (1.06) | 98.89 (3.53) | 0.73 (-2.29, 0.82) |
| PedsQL |  |  |  |  |
| Physical | 94.88 (7.88) | 92.19 (11.08) | 95.65 (6.77) | -3.46(-9.86, 2.94) |
| Emotional | 85.56 (17.43) | 83.13 (21.70) | 86.25 (16.42) | -3.13(-17.50, 11.25) |
| Social | 96.53 (8.27) | 96.88 (8.84) | 96.43 (8.26) | 0.45(-6.38, 7.28) |
| School | 82.78 (17.42) | 83.16 (20.34) | 82.68 (16.91) | 0.45(-13.95,14.85) |
| Psychosocial Health  Summary | 88.29 (11.40) | 87.71 (12.08) | 88.45 (11.42) | -0.74(-10.16, 8.67) |

**Participants with baseline FAI – Participants without baseline FAI*
